# Simulations using APSIM suggest that Conservation Agriculture sustains protein yield under changing climate dynamics in Northern Mozambique

**DOI:** 10.1186/s12870-025-07418-5

**Published:** 2025-11-13

**Authors:** Baqir Lalani, David Parsons, Mukhtar Ahmed, Uttam Kumar

**Affiliations:** 1 Natural Resources Institute, University of Greenwich, Medway Campus, Central Avenue, Chatham Maritime, Kent, ME4 4TB UK; 2 Department of Crop Production Ecology, Swedish University of Agricultural Sciences, UMEÅ, 90183 Sweden; 3 Department of Agronomy, Pir Mehr Ali Shah Arid Agriculture University Rwalpindi, Rawalpindi, 46300 Pakistan; 4 Department of Agroecology, Aarhus University, Tjele, 8830 Denmark; 5 Earth Systems and Global Change Group, Wageningen University and Research, Droevendaalsesteeg 3, Wageningen, 6708PB The Netherlands

**Keywords:** Conservation Agriculture, Climate change scenarios, Nutrition, APSIM, Crop diversity

## Abstract

**Background:**

Conservation Agriculture (CA) is based on the simultaneous practice of three principles: (i) no or minimum mechanical soil disturbance, (ii) permanent soil cover, and (iii) crop diversity e.g. crop rotation and/or intercropping systems. In parts of Sub Saharan Africa (SSA), conventional tillage practice is still pervasive and includes the practice of crop burning, resulting in severe soil erosion. Moreover, there is heavy reliance on maize, which contributes to limited dietary diversity. Crop modelling efforts allow for future scenarios to be explored to support policy formulation and farmer decision making. Research exploring potential benefits of CA on food and nutrition security has been limited and existing crop modelling efforts have failed to model the full CA system and/or have been limited to comparisons against monocultures or a narrow range of crops. The APSIM crop model was used to simulate the productivity and protein yield of a variety of intercropping systems involving three crops (maize, cowpea and pigeonpea) under full CA practice relative to conventional tillage (CV) with the same intercropping system. A baseline scenario used site-specific daily historical weather data acquired between 1997 and 2015 for Pemba-Metuge district in Cabo Delgado province (Northern Mozambique). A second set of simulations used incremental changes in temperature corresponding to future climate scenarios.

**Results:**

Results showed that temperature plays the most important role, contributing to nearly 60% of the variance in the combined protein yield. Projected trends further indicated that the combined protein yield of the three crops decreased from a median of 207 kg ha⁻¹ in the baseline scenario to 121 kg ha⁻¹ under a 4 °C temperature increase in the CV system. In the CA system, the median combined protein yield decreased from 230 to 135 kg ha⁻¹ under the same temperature scenarios.

Median grain yields declined from the baseline scenario to a 4 °C temperature increase by 267, 97, and 29 kg ha⁻¹ for cowpea, pigeonpea, and maize, respectively, under the CV system. Under the CA system, the corresponding declines were 291, 107, and 27 kg ha⁻¹. Nevertheless, protein yields and overall productivity remained consistently higher under the CA system.

**Conclusions:**

Our simulation work provides preliminary evidence that suggests Conservation Agriculture can sustain protein yield under changing climate dynamics in Northern Mozambique.

**Supplementary Information:**

The online version contains supplementary material available at 10.1186/s12870-025-07418-5.

## Background

Conservation Agriculture (CA) is based on the simultaneous practice of three principles: (i) no or minimum mechanical soil disturbance, (ii) permanent soil cover, and (iii) crop diversity e.g. crop rotation and/or intercropping systems (FAO, 2022). In parts of Sub Saharan Africa (SSA), conventional tillage practice, either with a hand-hoe or the use of animal traction, is still pervasive and includes the practice of burning crop residues, resulting in severe soil erosion and loss of soil organic matter (SOM) [1] (Rockstrom et al. 2009). Furthermore, in Southern Africa, although other crops are often grown, there is heavy reliance on maize (*Zea mays* L.) to fulfill food security needs rather than diversifying cropping systems [2] (Mhlanga et al. 2021). For example, in many parts of Malawi, farmers have reported that sole maize crop still occupies about a quarter of the land area cultivated, whilst between 50 and 60% is maize intercropped with one legume [3] (Mungai et al. 2016). The over-dependence on maize severely limits dietary diversity and results in nutritional deficiencies, a situation which is likely to worsen as a result of increased climate variability and climate change if no adaptation to cropping practices takes place [2] (Mhlanga et al. 2021). Moreover, the likelihood of increased extreme weather events as a result of climate change in the region has highlighted the importance of improving the adaptive capacity of farmers [4] (e.g. Morton 2007).

In recent years, the need for sustainable production intensification and diversified cropping systems that are ‘climate-smart’ [5] (Thornton et al. 2017) has gained momentum and the adoption of CA throughout the world (including parts of SSA) has increased substantially, estimated to be more than 180 million hectares [6] (Kassam et al. 2017). Wide scale benefits, including increased soil moisture, comparable or higher yield levels compared to those under conventional intensive tillage agriculture, improvement of biological processes (above and below the soil), and reduced soil erosion and leaching [6] (Kassam et al. 2017). Despite this, low rates of adoption of CA, particularly in Southern Africa, have contributed to some debate surrounding the benefits of CA for smallholder farmers in these regions [7, 8] (e.g. Giller et al. 2009; Giller et al. 2012). These include CA’s ‘climate smart’ properties (e.g. whether beneficial in dry or wet years) [9] (Michler et al. 2018), the benefits to yield particularly in the short-term, whether crop residue retention is viable given the trade-offs that exist with respect to feeding livestock and the extent to which CA leads to improvements in soil quality (Powlson et al. 2018) [10]. Whilst these aspects have been extensively debated, other authors have suggested a ‘niche’ exists where CA fits and this is likely to increase with time, particularly in Southern and Eastern Africa, given an increase in climate variability [11] (Baudron et al. 2015).

Use of ex-ante crop modelling approaches has been widely implemented in many higher income countries to examine different agricultural practices under future scenarios with respect to climate change and thereby support policy formulation and farmer decision making [12] (e.g. Morel et al. 2021) but have been ‘poorly integrated in SSA agricultural research’ [13] (Nhantumbo 2017). Whilst there has been previous research in the Southern Africa region and elsewhere that has simulated the use of CA [14–16] (Nhantumbo et al. 2016; Bahri et al. 2019; Roxburgh and Rodriguez 2016) this has been restricted to simulating the practice of CA with one or two crops [14–16] (Nhantumbo et al. 2016; Bahri et al. 2019; Roxburgh and Rodriguez 2016) and/or restricting it to specific comparisons of CA practices e.g. basins vs. direct seeding [17] (Nyagumbo et al., 2017). Previous research has explored the use of Climate Smart Agriculture (CSA) practices in Sub-Saharan Africa [18, 19] Zizinga et al., (2022; 2024) explored the impacts of CSA (e.g. planting basins and mulching) on maize productivity under present and future climate change scenarios in Uganda [20]. Olajire et al. (2022) assessed the impacts of indigenous CSA practices on yields of 3 staple food crops (maize, rice and cassava) in Nigeria (modelled separately) under a future climate (2031–2055) scenario [21]. Chimonyo et al., [2020] recently modelled the intercropping of maize with Bambara groundnut in South Africa using optimum management strategies (i.e. planting dates and plant populations) under future climate change scenarios. A recent systematic review, which assessed progress to date and gaps in crop simulation modelling research, with respect to climate change in Africa found the vast majority of studies focussed on staple crops (36% of studies) with very little focus on certain legumes (e.g. peanut) despite their value both economically and in terms of providing benefits to soil fertility, and even less priority has been given to neglected and underutilized crops such as Bambara groundnut, Amaranth, Cowpea, Cassava, Yam, Taro, and Lablab (1% of studies) [22] Benaly et al., (2025). Moreover [22], notes there is an urgent need for models that are locally calibrated and validated based on African agroecosystems. Crop models are invariably based on mono-crop systems, which fail to caputure the on-farm realities and complexity involving multiple crops [22, 23] (Benaly et al., 2025) (Chimonyo et al. 2015). Novel modelling approaches tackle Africa’s distinctive agricultural challenges, including intercropping and agroforestry systems, effectively capturing intricate interactions among crops and vegetation types [23] (Chimonyo et al. 2015). Such localised calibration involves the need for regional data, including on specific agricultural practices and soil thereyby enhancing the accuracy of modelling outputs [24] (Choruma et al. 2019).

Furthermore, research exploring the ‘potential synergistic benefits’ of CA on nutrition is scanty and particularly important in the Southern African context given the reliance on maize and the likelihood of increasing climate variability due to climate change [2] (Mhlanga et al. 2021). Within Northern Mozambique (Pemba-Metuge district in Cabo Delgado province) which is the focus area of this study, although maize still dominates, maize-legume systems are commonplace. Cowpea (*Vigna unguiculata* Walp) and pigeonpea (*Cajanus cajan*) are commonly grown [13] (Nhantumbo 2017) either as intercrops or as a sole crop. Whilst there has been some debate surrounding whether legume productivity fares better within an intercrop rather than as a sole crop [13] (e.g. Nhantumbo 2017) more research is being done on identifying adequate cropping sequences in legume-cereal cropping systems across Africa. For example, for other countries in Africa such as in Ghana, maize yields of up to 7.0 t/ha were measured from a pigeonpea-maize sequence, compared to 2.0 t/ha measured in a continuous maize system [25] (Adjei-Nsiah, 2012).

There has been no research to date that has explored ex-ante application of full CA practice (i.e. use of all three principles and at least three different crops as defined by [26] FAO (2022)) and particularly within the context of Northern Mozambique, on crop yield or protein yield under a variety of climate-change scenarios. We therefore test the hypothesis that Conservation Agriculture contributes to improvements in grain yield and protein yield under changing climate dynamics relative to conventional tillage-based agriculture.The novelty of our approach is further enhanced by our focus on underutlised crops^1^ such as cowpea and pigeonpea;^2^ exploration of multicrop interactions/locally specific agricultural practices and localised calibration of weather and soil data thereyby enhancing the accuracy of modelling outputs [22–24]

The objectives of this study are to investigate how the full CA practice under various climate change scenarios (changes in temperature and rainfall) affects protein yield relative to conventional-tillage based agriculture (CV) for a variety of intercropping systems involving three crops (maize, cowpea and pigeonpea**)**, using a simulation modelling approach.

## Methods

### Site description-Cabo Delgado

Mozambique consists of ten different agro-ecological zones. These have been grouped into three different categories which are based in large part on mean annual rainfall and evapotranspiration (ETP) [27] (INIA, 1980). The relative importance given to various crops reflects a high correlation with the agro-ecological context with food and cash crops.

**Fig. 1:**
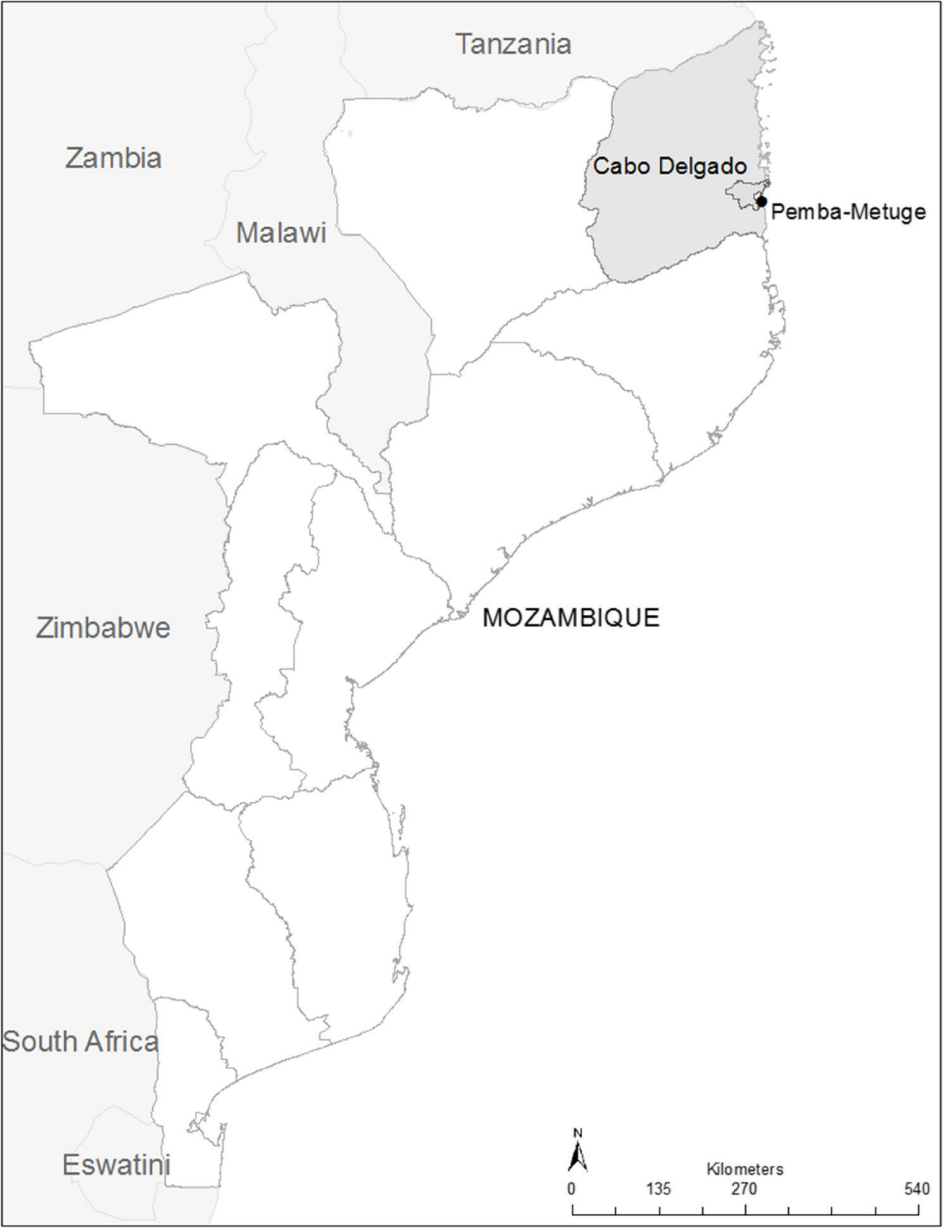
Map of Mozambique showing the studied province (Cabo Delgado, in grey) and district (Pemba-Metuge, see black dot and outlined within the province)

The low altitude zones in the country (R1, R2, R3, R5, R6, R7, R8) are typically hot with comparatively low rainfall (< 1000 mm mean annual rainfall) and high ETP. In contrast, the medium altitude zones (R4, R7) have a mean annual rainfall ranging between 900 and 1500 mm and medium level of ETP. The highland areas of Mozambique, however, are typified by high rainfall regions (> 1000 mm, mean annual rainfall) with low evapotranspiration (R3, R9 and R10). The Cabo Delgado province (Fig. 1) falls within three agro-ecological zones R7, R8 and R9. The particular district under study (Pemba-Metuge within Cabo_Delgado province) is situated within the R8 agroecological zone (Fig. 1) (for a more detailed description of the agroecological zones in Mozambique see [28] (Silici et al. 2015) where distribution of rainfall is often variable with many dry spells and frequent heavy downpours.

The predominant soil type in the R8 agroecological zone is Alfisols [29] (Maria and Yost 2006) typified by red clay soils which are typically deficient in nitrogen and phosphorous [30] (Soil Survey Staff 2010). Furthermre, Cabo Delgado ranks as the second poorest province in Mozambique [31] (INE 2012) and has one of the highest rates of stunting prevalence in the country [32] (Fox et al. 2005). The district of Pemba-Metuge, in the province of Cabo Delgado, Mozambique (Latitude 13° 01’26.47’’ S and Longitude 40° 23’35.72” E) has a sub-humid climate (moist Savanna) characterised by two seasons, namely one season which is considered the dry season (May to November) and the other wet season (December to April). The district of Pemba-Metuge has an average annual precipitation of 861 mm and an average annual temperature of 25°C. Maize is generally grown under rain-fed conditions, with limited use of purchased inputs such as improved seeds, pesticides and inorganic fertilisers. Irrigation is concentrated mostly along the river valleys in the southern part of Mozambique. Maize is largely grown as a subsistence crop and it is often cultivated as a dominant intercrop alongside grain legumes such as cowpea, beans, groundnuts and pigeonpea.

### Crop modelling

APSIM has been designed with a modular structure and users can select and integrate different components (e.g., soil water, crop growth) depending on the specific agricultural system being studied. This flexibility allows for a tailored approach to modeling different crops, climates, and management practices. Many crop models are either too simplistic or too specific to particular crops or regions. APSIM’s modularity and wide range of crop models make it versatile and adaptable to many different agricultural systems globally. APSIM simulates crop growth based on a detailed representation of key physiological processes, such as photosynthesis, respiration, nutrient uptake, and water use. It also incorporates the effects of climate, soil properties, and management practices like irrigation and fertilization. Compared to more simplified models, APSIM provides a more realistic and mechanistic understanding of crop performance. This is especially useful when precision is required for investigating different management scenarios (e.g., fertilizer rates or irrigation schedules) or under varying environmental conditions. Simulations of plant growth and development were performed using the mechanistic crop model APSIM (version 7.10). APSIM simulates crop growth and development on a daily basis as a function of climatic conditions and agronomic inputs [33] (Holzworth et al. 2014). Crop development is simulated using a thermal time approach to identify different phenological stages and phases. Leaf area index intercepts the radiation which is converted into biomass by multiplying the radiation interception with the radiation use efficiency factor [34] (Asseng et al. 2000). Furthermore, temperature, water and N stress affect biomass availability for crop growth as shown in flow diagram (Supplementary Fig. 1 A & B). It is then partitioned into leaf, stem, roots and pods (grain) based on growth stage and partitioning factors. Depending on the available biomass for grain development, grain yield is simulated by interacting parameters regulating kernel number, grain growth and grain filling rate [35] (Keating et al. 2003). More description of the general crop mechanisms in the model can be found online (https://www.apsim.info/wp-content/uploads/2019/09/WheatDocumentation.pdf).

### Calibration of the APSIM-Maize, APSIM-Cowpea and APSIM-Pigeonpea models

APSIM-Maize was calibrated by modifying the parameters of the default cultivar Katumani in APSIM with data obtained from different published articles [36, 37] (e.g. Magaia et al., 2017; Magaia, 2017). The article by [38] Harrison et al. (2011) was the primary source of data used to calibrate maize crop phenology. Flowering 42 days after sowing and maturity 90 days after sowing was used to calibrate all maize crop phenology coefficients. Similarly, leaf area was calibrated using previous published articles from the study region [39, 40] (Chimonyo and Mabhaudhi, 2016a; Chimonyo et al. 2016b). Yield data from [41] FAOSTAT (2023) was used to calibrate maize yield (i.e. historical data available for maize and cowpea yields for Mozambique for the years 1997–2015). Historical pigeonpea yields were not available for Mozabique from FAOSTAT and therefore yields based on ‘expert’ opinion/researchers based in the region were used (Jose Dambiro, personal communication) and adjusted in order to be more representative of the region (e.g [42]. USAID et al. 2017; Jose Dambiro, personal communication) and to account for shorter duration varieties that were being used or experimented within the region (e.g [43]. Cachisso Bambo Donça et al. 2021: Jose Dambiro personal communication, 2018). Calibration was initially performed for all treatments. For calibration, cultivar coefficients were obtained step by step, first for parameters regulating phenological development and then for grain development, as suggested in [44] Seidel et al. (2018). The manual trial and error method was used to determine genetic coefficients [45] (Godwin and Singh 1998). Parameter values were adjusted to have minimum skill score e.g. root mean square error (RMSE) between simulated and observed data on flowering and physiological maturity date, leaf area index (LAI), biomass and yield. The same values of this set of parameters were used to evaluate performance and robustness of the APSIM-Maize model calibration. The same procedure was repeated to calibrate and evaluate the APSIM-Cowpea and Pigeonpea models. Calibrated values of the parameters are presented in Table 1.

**Table 1:** Calibrated parameters for APSIM-Maize, APSIM-Cowpea and APSIM-Pigeonpea

Name	Unit	Maize	Cowpea	Pigeonpea
Photop_sens (Photoperiod Sensitivity)	-	-	-	-
Vern_sens (Vernalisation Sensitivity)	-	0	0	0
RUE (Radiation use effeciency)	g/MJ	1.6	2.1	1.1
tt_emerg_to_endjuv (Thermal time needed from emergence to end of juvenile)	°C	50	450	300
tt_endjuv_to_init units (Thermal time needed from end of juvenile to floral initaitaion)	°C	179	35	50
tt_flower_to_start_grain (Thermal time needed from flowering to start grain filling)	°C	180	50	33
tt_flower_to_maturity (Thermal needed from flowering to maturity)	°C	879	600	567
potKernelWt (Potential kernel weight)	g/m^2^	350	-	-

### Soil data

Soil physical and chemical characteristics were measured using laboratory methods and using existing datasets. For Cabo Delgado (Metuge district), soil data based on soil sampling conducted within the district (i.e. from experienced CA and conventional farmers in 2013/2014 by the lead author and Jose Dambiro (Personal communication, Aga Khan Foundation and Jose Dambiro) were used to compute an estimation of the soil water characteristics using the SPAW (Soil-Plant-Air-Water) model [46] (Saxton and Rawls 2006). Soil samples were collected from four experienced CA farmers (using CA for at least 5 years or more) and one farmer that had never practiced CA from their main plot of land in Pemba-Metuge District before harvest, in May/June 2013. For each field, five cores were taken (in a zig-zag format) and mixed to obtain a unique composite sample, for two different depths (i.e. 0–20 and 20–40 cm). Soil samples were then analysed at the ARC-Institute for Soil, Climate and Water in Pretoria (South Africa). Composite samples were analysed for texture, pH (water), organic carbon (loss on ignition), total nitrogen (Kjeldhal method), available phosphorus (Bray method) and available potassium (ammonium acetate extraction). In addition, five undisturbed cores at two different depths were taken for each field (main plot of land) in order to measure bulk density. Tables 2 and 3 highlight the APSIM parameterization for the soil physical characteristics and specific values used.

**Table 2:** Soil physical and chemical characteristics used for APSIM parameterization

Depth (cm)	Sand (%)	Silt (%)	Clay (%)	Texture	pH	NO_3_ ^−1^ (Kg/ha)	NH_4_^+^ (Kg/ha)	OC (Total %)
0–10	22	52	26	silty loam	6.5	27.6	5.52	1.34
10–30	22	52	26	silty loam	6.5	28.4	5.68	1.10
30–60	22	52	26	silty loam	6.5	21.9	4.38	0.67
60–90	22	52	26	silty loam	6.5	22.5	4.5	0.34
90–120	22	52	26	silty loam	6.5	22.8	4.56	0.20

**Table 3:** Details of soil water parameter values used for APSIM simulations

Depth (cm)	BD (g/cc)	Air Dry (mm/mm)	LL15 (mm/mm)	DUL (mm/mm)	SAT (mm/mm)	Ks (mm/day)	Maize LL (mm/mm)	Maize PAWC	Maize KL (/day)	Maize XF (/day)	Cowpea LL (mm/mm)	Cowpea PAWC	Cowpea KL (/day)	Cowpea XF
0–10	1.38	0.168	0.168	0.339	0.479	195.12	0.168	17.1	0.08	1	0.168	17.1	1	1
10–30	1.42	0.166	0.166	0.334	0.464	162.72	0.166	33.6	0.08	1	0.166	33.6	1	1
30–60	1.46	0.163	0.163	0.328	0.448	123.36	0.163	49.5	0.08	1	0.163	49.5	1	1
60–90	1.5	0.160	0.160	0.323	0.432	95.04	0.160	48.9	0.08	1	0.160	48.9	1	1
90–120	1.52	0.159	0.159	0.32	0.426	86.64	0.159	48.3	0.06	1	0.159	48.3	1	1

*BD* Bulk desnsity, *LL15* Lower Limit or wilting point (-15 bars), *DUL* Drained Upper Limit or field capacity (-0.33 bar), *SAT* Saturated soil (0 bars), *Ks* Gravitational flow after all soil pores are filled up, *PAWC* Plant Available Water Content, *XF* Root exploration factor (1 means unlimited exploration), *KL* Fraction of PAW able to be extracted

### Protein yields

To evaluate the combined protein value of each cropping system, we included grain yield of all the crops in the cropping system (e.g. maize and the legume) and calculated protein yield (kg ha^−1^). Values for grain protein concentrations were obtained from [47] Gulzar and Minnaar (2017) for pigeonpea and cowpea (e.g. 21.7 g per 100 g for pigeonpea and 23.85 g per 100 g for cowpea) and values from [48] Meier et al. (2020) were used for maize (9.4 g per 100 g). 3

### Farming practices and treatments used

Farming practices were defined for each crop based on local expert knowledge (Table 4). Sowing and cut-off harvest dates were defined based on usual practices for each crop. The specific treatments (including sowing dates, plant densities and depths) are shown in Table 5. Irrigation or the incorporation of any other inputs such as fertiliser, manure/compost, herbicides or pesticides were not used in the simulations, in order to better reflect the farmer realities in the study region. Furthermore, sowing was considered ‘on the flat’ for CA since the model does not have the option for the use of basins. Additionally, stubble burning is a usual practice in farmer fields but it was not used in the model (Table 5). Crop residue was retained in CA and removed for conventional tillage. No weeds were included in the simulation.

**Table 4:** Common farming practices and intercropping systems under conventional and Conservation Agriculture that were used to prepare simulation setups

Crop diversity	Conventional Agriculture	Conservation Agriculture
Non diverse	Tillage Intercrop: Maize and cowpea	No tillage. Mulch. Intercrop: Maize and cowpea
Diverse	Tillage Intercrop: Maize, cowpea, pigeonpea	No tillage. Mulch. Intercrop: Maize, cowpea, pigeonpea

**Table 5:** Sowing and harvest details of crops in intercropping systems under Conservation and Conventional Agriculture

Conservation Agriculture (CA)							
	Crop	Sowing window	Sowing density (plant/m2)	Sowing depth (mm)	Row spacing (mm)	Type of variety	Harvest cut off
Diverse, Non-Diverse	Maize	24 Nov − 15 Jan	6	20	800	Early (90 DAS) Katumani	31-Mar
Diverse, Non-Diverse	Cowpea	24 Nov − 15 Jan	12	20	40	Medium (85 DAS) Banjo	30-May
Diverse, Non-Diverse	Pigeonpea	24 Nov − 15 Jan	2	30	800	Medium (180 DAS) Extra short duration	31-Aug
Conventional Agriculture (CV)							
Diverse, Non-Diverse Diverse, Non-Diverse	Maize	24 Nov − 15 Jan	6	20	800	Early (90 DAS) Katumani	31-Mar
Diverse	Cowpea	24 Nov − 15 Jan	15	20	40	Medium (85 DAS) Banjo	30-May
Non-Diverse	Cowpea	24 Nov − 15 Jan	8	20	40	Medium (85 DAS) Banjo	30-May
Diverse	Pigeonpea	24 Nov − 15 Jan	2	30	800	Medium (180 DAS) Extra short duration	31-Aug
Non-Diverse	Pigeonpea	24 Nov − 15 Jan	2	30	500	Medium (180 DAS) Extra short duration	31-Aug

### Model application: description of historical dataset and climate change scenarios tested

**Fig. 2:**
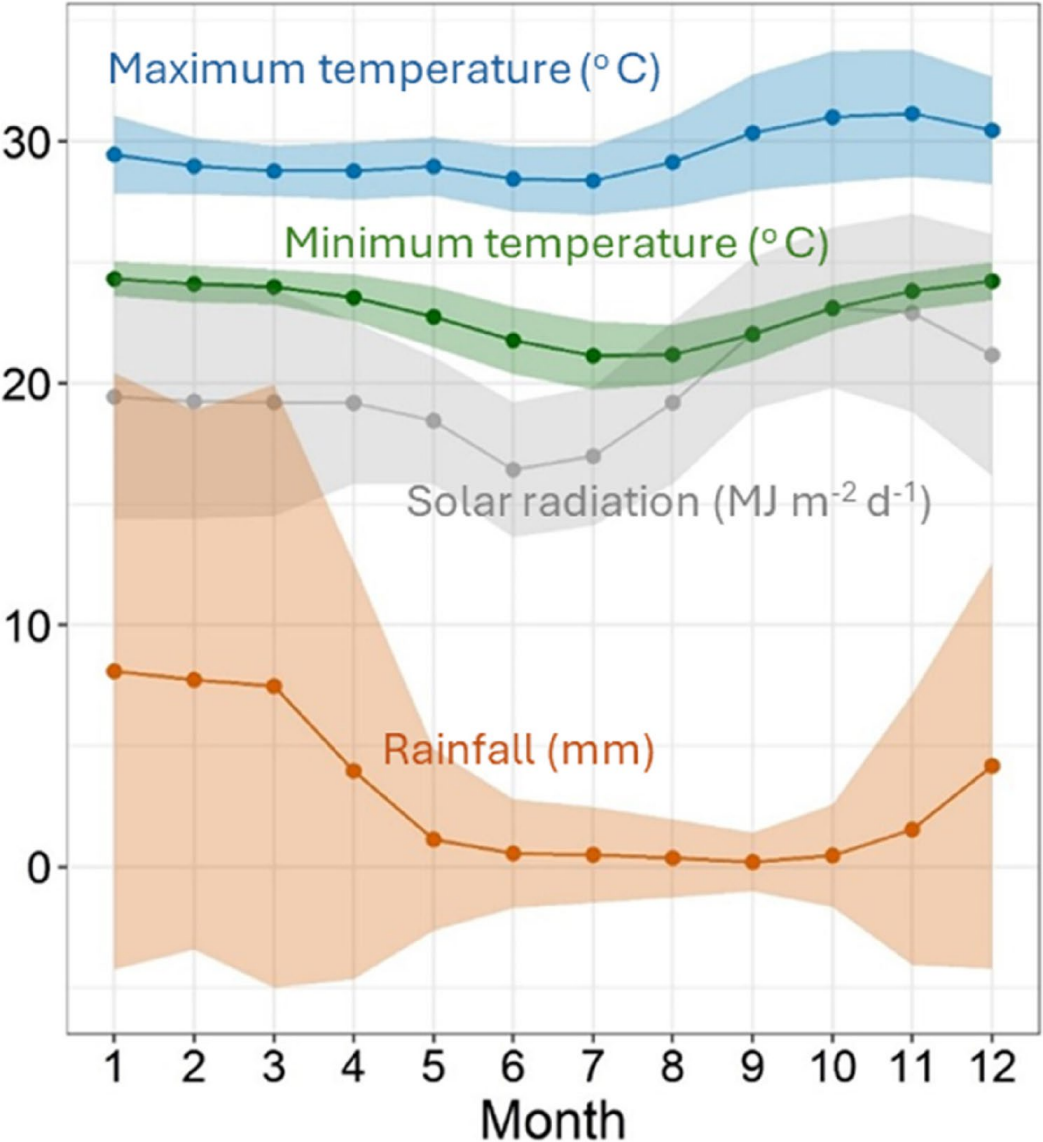
Monthly mean and standard deviation of historical climate for 1997–2015. Maximum temperature (°C, Mean = blue dots and line, standard deviation = blue shaded area), minimum temperature (°C, Mean = green dots and line, standard deviation = green shaded area); solar radiation (MJ m^−2^ d^−1^, Mean = gray bar, standard deviation = gray shaded areaerror bar); rainfall (mm, Mean = orange dots and line, standard deviation = orange shaded area)

Historical climate data for the Pemba-Metuge district (latitude= −12,974; longitude = 40.390) were sourced from CCAFS (http://www.ccafs-climate.org). Climate data, including rainfall, minimum temperature and maximum temperature and solar radiation were obtained for the period 1997–2015 (Fig. 2).

Climate scenarios were generated using the factorial tool in APSIM by increasing historical daily temperature by 0, 1, 2, 3 and 4 °C and rainfall by 0, 10 and 20% while keeping CO_2_ concentration the same (360 ppm). All intercropping systems under CV and CA settings were simulated in APSIM (Table 4). The combination of daily increase in temperature and rainfall is within the range of predicted mean temperature and rainfall towards the end of the century (2080–2099) from the reference period of 1995–2014 based on the Shared Socio-economic Pathways (SSPs) 1-1.9 to 8.5 (https://climateknowledgeportal.worldbank.org/country/mozambique/climate-data-projections). SSPs 1-1.9^4^ is based on the economic growth model towards a more sustainable path while SSPs 8.5 is based on a high fossil-fuel usage pathway. Similar approaches of incremental or factorial combinations of increased temperature and rainfall from historical data were used in earlier studies to assess the effects of climate change on crop yield and variability using APSIM [12, 49](Morel et al. 2021; Lobell et al. 2013).

**Table 6:** Calibration of crop phenology and grain yield shown with RMSEs along with other relevant variables incorporate: MAE: Mean Average Error, NSE: Nash-Sutcliffe Efficiency

Crop	Flowering	Maturity	biomass (kg/ha)	yield (kg/ha)
	DAS (das)	DAS (das)		
Maize	42 (RMSE = 2.79 d, MAE = 2, Bias = 1.6 and NSE = 0.02)	92 (RMSE = 8.30 d, MAE = 2.2, Biass = 0.33 and NSE = 0.16)	6420 (RMSE = 394 kg/ha, MAE = 14.2, Biass = 1202 and NSE = 0.61)	1365 (RMSE = 105 kg/ha, MAE = 13.8, Biass = 84 and NSE = 0.31)
Cowpea	40 (RMSE = 3.43 d, MAE = 1.6.89, Bias = 0.5 and NSE = 0.03)	66 (RMSE = 5.30 d, MAE = 1.7, Biass = 0.22 and NSE = 0.07)	2130 (RMSE = 143 kg/ha, MAE = 7.3, Biass = 0.23 and NSE = 0.09)	519.7 (RMSE = 70.3 kg/ha, MAE = 9.1, Biass = 0.26 and NSE = 0.17)
Pigeonpea		Short duration between (< 105 days (Snapp et al., 2003)^b^		0.2–385 kg/ha (USAID, 2017, Degrande 2001)^a^

^a^We adjusted yields slightly lower given small plots of land and reflecting that it is the poorest region in Mozambique (See USAID, 2017)

^b^extra-short/short duration types were starting to be experimented with by NGOs/farmers and researchers in the region. See Cachisso Bambo Donca et al. 2021; Dambiro, Personal communication (2018)

### Statistical analysis

To analyse the effects of a system, temperature and rainfall on the yields of individual crops and their combined protein yield, linear effect models were constructed using the *lm* () function from the R base package [50] (R Core Team 2021). The models were constructed using system, temperature and rainfall as fixed effects. Data transformation was applied due to non-normality: log_10_ transformation was applied for yields of cowpea, pigeonpea and combined protein yields of the crops. The 4th order exponential form was used to transform maize yield for the analysis. The analysis was performed in R Studio (version 1.4.1106) [51] (R Studio Team 2020).

Sum of Squares (SSQ) and Mean Sum of Squares (MSE) in the analysis of variance output from linear effect models were used to compute the proportion of variance. The proportion of variance (ω^2^) explained by each factor was computed using the following equation (based on [52] Dodd and Schultz, 1973).

$$\omega^2=\frac{SSQ_{vi}-\left(n-1\right)MSE}{SSQ_{total}+MSE}$$

Where $\:{SSQ}_{vi}$ is the sum of squares of the variance for the factor or interaction, $\:n$ is the number of unique cases under factor or interaction, $\:{SSQ}_{total}$ total sum of squares of the variance and $\:MSE$ is mean square error.

## Results

### Crop calibration

Crop calibration in APSIM for maize, cowpea and pigeonpea for phenological, biomass and grain yield related parameters were robust and based on the relative root mean square error (RRMSE: RMSE/mean x100). The RRMSE was between 6.6 and 9.0 for phenology, 6.1–6.7 for biomass, and 7.7–13.5% for grain yield for maize and cowpea. For pigeonpea, the phenology and grain yield were adjusted to be representative of small holdings and current experimentation with short duration varieties. Therefore, RMSEs were not computed for pigeonpea calibration. Together with RMSEs, MAEs, Bias and NSEs are also presented in Table 6.

### Protein Yield under CA and Conventional Agriculture

**Fig. 3:**
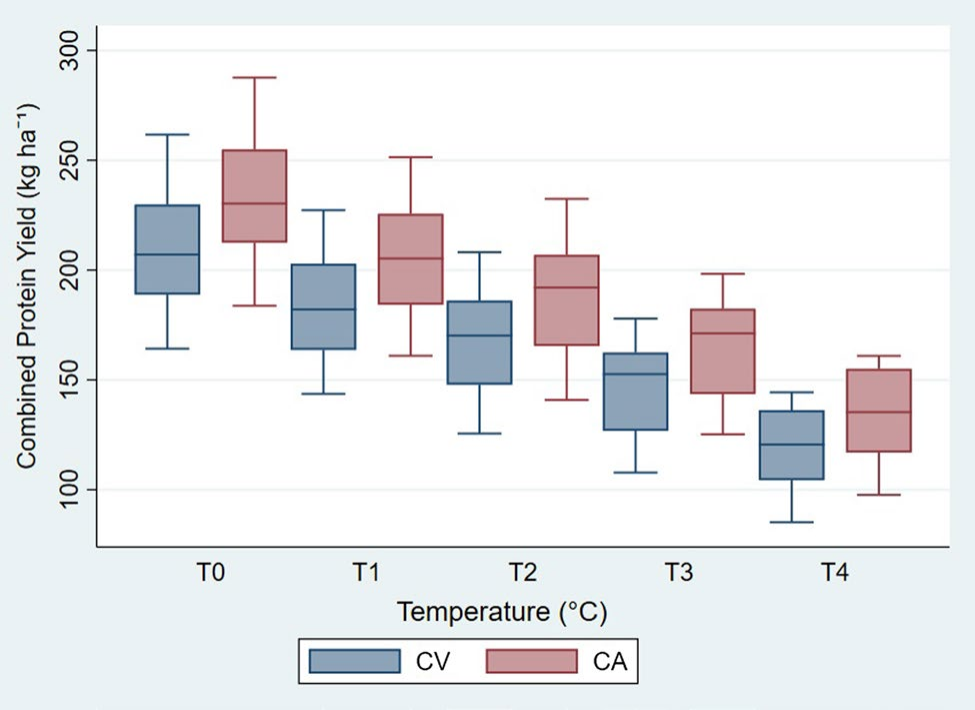
Combined protein yield (kg ha^−1^) under Conventional Agriculture (CV) and Conservation Agriculture (CA) with increasing temperature (T0 to T4: from 1 to 4 °C). Blue box-plots denote CV and red CA Please note that Figures 3 to 5 include observations from the three-crop intercropping system under different temperature scenarios

**Fig. 4:**
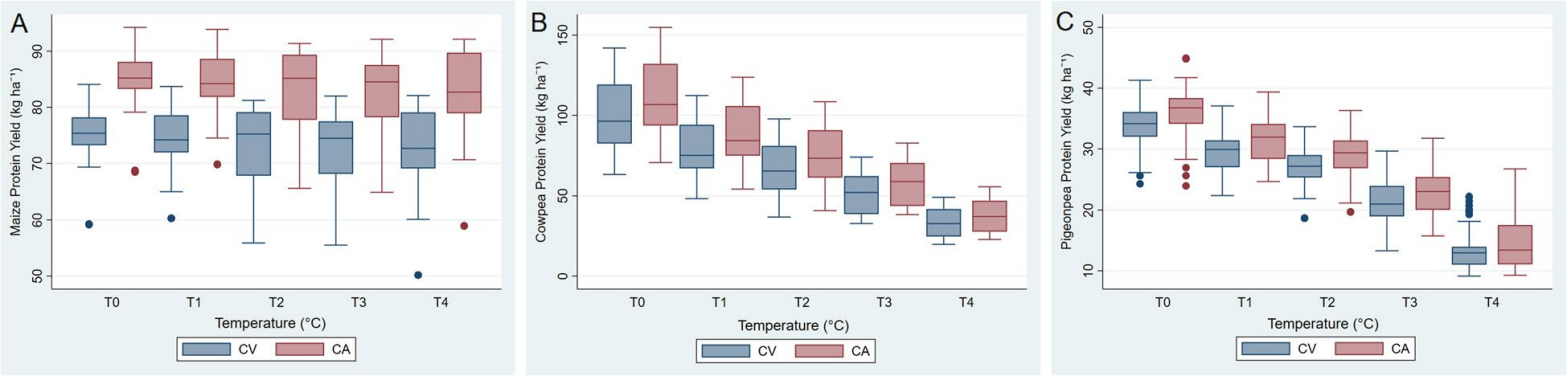
Protein yield (kg ha^−1^) of (**A**) maize, (**B**) cowpea and (**C**) pigeonpea under Conventional Agriculture (CV) and Conservation Agriculture (CA) with increasing temperature (T0 to T4: from 1 to 4 °C). Blue box-plots denote CV and red CA

Temperature was the primary factor that affected the combined protein yield (Table 7), contributing close to 60% of the variance. Under both conservation and conventional systems, combined protein yield consistently declined with increasing temperature (Fig. 3). The combined protein yield of all crops under CA was higher than under CV, which was more visible for maize and cowpea and less for pigeonpea (Fig. 4A and C). Protein yield was highest for cowpea and lowest for pigeonpea, with no increase in temperature from historical climate under both systems of agricultural practices. However, with the 4 °C increase in temperature, maize has the highest protein and pigeonpea the lowest. Changes in rainfall did not affect protein yield.

**Table 7:** Proportion of variance (ω^2^) explained by the factors for yield of individual crops and combined^a^ protein yield. Conventional agriculture (CV) and Conservation Agriculture (CA)

Factor	Combined protein yield (ω^2^, %)	Maize yield (ω^2^, %)	Cowpea yield (ω^2^, %)	Pigeonpea yield (ω^2^, %)
System (CV and CA)	5.89	20.93	2.70	0.85
Temperature	58.00	1.35	59.8	76.36
Rainfall	−0.06	−0.13	−0.07	−0.02
System: Temperature	−0.12	−0.27	−0.13	−0.14
System: Rainfall	−0.06	−0.14	−0.07	−0.09
Temperature: Rainfall	−0.26	−0.55	−0.27	−0.31
System: Temperature: Rainfall	−0.26	−0.56	−0.27	−0.33

^a^Combined refers to the combined protein value of all the crops in the intercropping system i.e maize, cowpea and pigeonpea

### Crop yield under CA and conventional with climate change factors

**Fig. 5:**
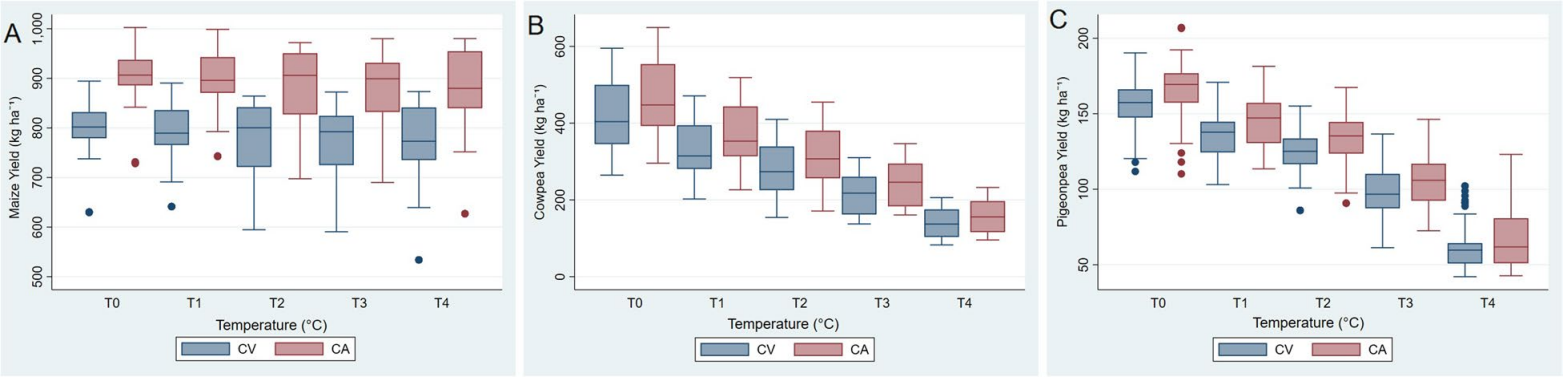
Grain yield (kg ha^−1^) of (**A**) maize, (**B**) cowpea and (**C**) pigeonpea under Conventional Agriculture (CV) and Conservation Agriculture (CA) with increasing temperature (T0 to T4: from 1 to 4 °C). Blue box-plots denote CV and red CA

The systems i.e. conservation and conventional agricultural, contributed primarily to the variance in maize yield (Table 7). Yield was higher under CA compared with CV (Fig. 5). Temperature change was the second largest source of variance in maize yield.

In contrast to maize yield, the contribution of temperature to the variance in cowpea and pigeonpea yield was the primary factor (Table 7). Yield of both crops had a declining trend with increasing temperature (Fig. 5b and c). Similar to the yield of maize, yield of cowpea and pigeonpea was higher under CA than CV (Fig. 5b and c); however, the system only explained a small proportion of the variance. Moreover, protein yield and productivity differed only slightly between diverse and non-diverse intercropping systems under both CV and CA. 

**Fig. 6:**
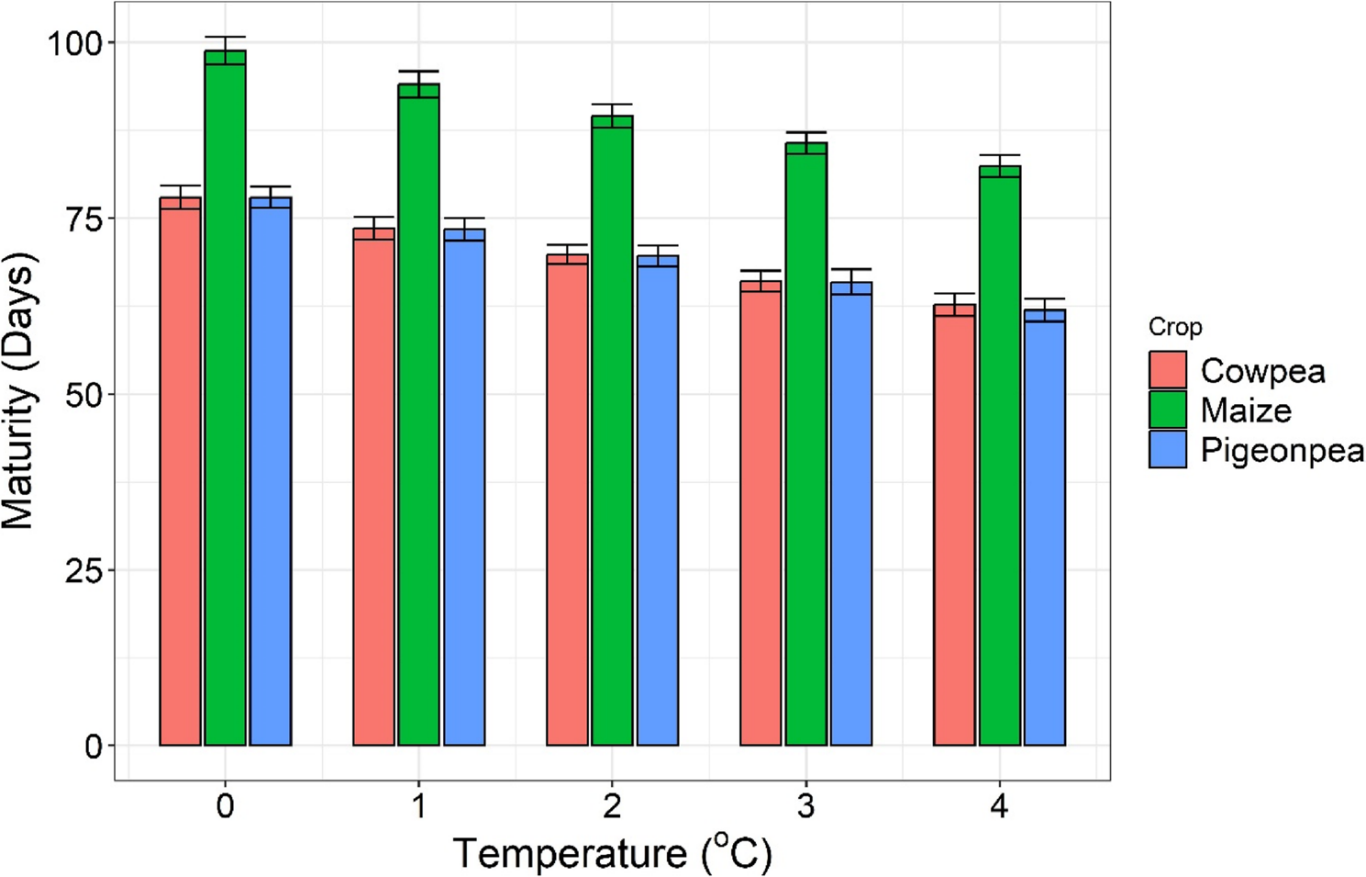
Days to maturity of crops for four temperature treatments across years (1997–2015), Conservation Agriculture (CA) and Conventional agriculture (CV) with mean and standard deviation

Days to maturity decreased by 15–17 days, depending on the crop, as temperature increased from 0 to 4 °C (Fig. 6). Decrease in days to maturity with increasing temperature was observed for all the three crops.

## Discussion

Maize dominates cultivated areas in several Southen African regions [2, 3] (Mhlanga et al. 2021; Mungai et al. 2016). There is a dietary diversity limitation in the region which poses food and nutritional security challenges under future climatic conditions [4](Morton 2007). In Northern Mozambique, maize is the dominant crop though it is also grown in a maize-legume intercropping system on many farms [13] (Nhantumbo 2017). In this study, we evaluated the potential of diversification of maize-based intercropping systems with the inclusion of cowpea and pigeonpea and examined their response to current and future climate with respect to overall yield and protein yield under CV and CA in Northern Mozambique. Higher yield and combined protein yield under CA compared to CV systems (Fig. 3) suggest that the results are in line with reported benefits of CA such as preventing soil erosion [53], (e.g. Kassam et al., 2009) soil moisture conservation [54] (Ngwira et al. 2014), improved resource use efficiency, as well as sustaining long-term crop productivity [55] (Gwenzi et al. 2009), hence helping crops to perform better than under a CV system.

The results also indicate that increasing temperature has a detrimental impact on overall yield and combined protein supply for both maize-cowpea and maize-cowpea-pigeonpea intercrops under CV and CA systems (e.g. Table 7). Protein yield and yield of cowpea and pigeonpea declined steadily with increasing temperature; though this was less so for maize (Fig. 4). One reason that maize is likely to perform better than other crops in conditions with increased risk of drought is that the root system can penetrate soil deeper, which allows maize to extract water from the deeper soil layers [12] (Morel et al. 2021). In addition, other research in SSA has found that under CA there have been improvements of maize yield and maize has shown resistance to drought stress, especially at anthesis [56] (Steward et al., 2019).

Better performance of maize under drought conditions can heuristically be linked to its C4 photosynthetic mechanism. RuBisCO binds oxygen instead of CO_2_ with the C3 photosynthetic mechanism under high temperature and drought, which lowers the photosynthetic efficiency compared with C4 crops [57] (Sage et al. 2010). Because of this mechanism, maize performs at a higher optimum photosynthetic temperature and uses less water to fix each molecule of CO_2_ in comparison to C3 crops [58, 59] (Dwyer et al. 2007; Correia et al. 2021) such as cowpea and pigeonpea. Our results show that protein yield and productivity were not very different when temperature increased from 0 to 4^°^C in both systems. This indicates that the model captured the effect of the C4 mechanism under increasing temperature in both systems. A reported 57% decrease in maize yield with an increase of temperature by 3.3^o^ C in Mali has been shown in a modelling study using APSIM [60] (Traore et al. 2017) which is in contrast to our results where yield is less affected with an increase of temperature by 4^o^ C. However, the 1 to 8% mean decrease in maize yield observed in our study (with a median decline from 802 to 773 kg ha^-1^ for CV, and from 907 to 880 kg ha^-1^ for CA, as shown in Fig. 5A) under both systems is lower than the 10–20% yield decline reported for sub-Saharan Africa during 1980–2010 in response to climate variability [61] (Rosenweig et al., 2014), a decrease of 20% with 4 °C increase in temperature relative to 1980–1999 [62] (Thornton et al., 2011) and a decrease of 20% by 2045–2065 relative to climate change during the 1961–2002 period (Schlenker and Lobell 2010) [63]. Variations in maize yield under climate change studies are large (e.g. −98% to + 16%) [64, 65] (Fischer et al., 2001; Parry et al. (2004). Nevertheless, similar to the results in our study, most studies have predicted a negative change [66] (Challinor et al., 2007). These variations in maize yield could arise from many factors, for example, the differences in geographical area, year to year variation, inputs of soil properties, the use of global climate model (GCM) data, and historical cli­mate data that affect the soil water dynamics and vapor pressure deficits and evapotranspiration.

Pigeonpea is a drought tolerant crop that can withstand drought better than other legumes [67, 68] (Valenzuela and Smith, 2002; Okiror, 1986) due to its deep rooting system [69] (Flower and Ludlow, 1987) and better osmotic adjustment (OA) in the leaves under stress conditions [70] (Subbarao et al., 2000).

In our study, the grain yield of pigeonpea declines by more than 50% under both CV and CA systems with 4 °C increase in temperature relative to the climate from 1997 to 2015. This decline is higher than a reported decline of 3%, with an increase of 3.1 °C by the end of the century in Kenya [71] (Dimes et al.). The study is not exactly comparable since Dimes et al. considered 700 ppm of CO_2_. Nevertheless, with limited literature for Mozambique and the region, from a contextual perspective, the Dimes et al. study reports similar findings to our study that temperature is the most important factor in yield decline in future climatic conditions.

Similarly, modelling studies have reported that grain yield of cowpea would decline by 30% by 2025 compared to the climate for the period from 1951 to 1998 in Niger and 17% in Kenya with a 3 °C increase in temperature from the weather conditions of an experimental period from 2010 to 2011 [72] (Duivenbooden et al. 2002). The major cause of decline with 23% of grain yield in Kenya was rainfall, contrary to no effect of rainfall in our study. This could be related to the rainfall distribution between Kenya and Mozambique and the use of a short climate reference period as opposed to large reference periods in our study.

Maturity of maize was reduced on average by 17 days, with the temperature increase of 4 °C; however, protein yield was slightly lower (Fig. 5). These results are also in line with a crop modeling study using APSIM [73] (Tao and Zhang 2010), which reported a greater impact of increasing global mean temperature on maize phenology than on productivity in China. The steeper decline of protein yield of pigeonpea and cowpea with increasing temperature is probably due to the combined effect of fewer days for photosynthesis and higher photorespiration than maize.

Similarly [74], Rurinda et al. (2015), using APSIM for two contrasting sites in Zimbabwe (where temperature is projected to increase without any significant change in mean annual rainfall) under two radiative forcing scenarios and several time periods, found decline in maize yield with more significant yield declines in maize cultivars found in the long run and with high radiative forcing scenario compared to low scenario. More importantly, the modelling further identified the limited role of altering management options such as planting date and the need to transform the current cropping systems of southern Africa to offset the negative impacts of climate change. Seyoum et al. [75] (2017) further used the APSIM model to characterise major drought patterns and their frequencies experienced by maize cropping systems in the target population of environments spread across six countries of the region including Ethiopia, Kenya, Tanzania, Malawi, Mozambique and Zimbabwe. The authors identifying that the maize crop might experience stress in 54% of the seasons in the region though the frequency and pattern of drought varied with respect to patterns varied in relation to locations, genotypes and management.

Traore et al. [60] (2017) further identified that due to future climate and current cropping practices, food availability is expected to reduce for all farm types in southern Mali. More importantly, the research highlighted the need to account for the diversity of farmers in the region and that crop management strategies must be tailored to the capacity and resource endowment of local farmers.

Notwithstanding this and system differences, our findings support those of “doubled-up” rotations (i.e. two legumes with complementary phenology are intercropped and grown in rotation with maize) which have shown to improve overall protein yield (though these were modelled with a range of N fertilizer rates albeit yield benefits were found without increasing fertilizer inputs). Previous research has also found that the integration of leguminous crops results into higher nutritional value either by intercropping or rotating grain legumes with maize [77, 78] (Snapp et al., 2002;Mupangwa et al., 2021). These findings support the notion of increasing diversity into maize based systems, particularly given that imbalanced diets due to excessive consumption of carbohydrates such as maize causes poor dietary diversity in current cropping systems [79] (Akombi et al. 2017) which have been associated with stunting and wasting [80] (Murendo et al. 2018). Furthermore, staple foods such as maize may increase energy availability but they do not improve nutritional outcomes [81] (e.g. Rajendran et al. 2017).

Smith et al. [82] (2016) showed that doubled-up legume rotations improve soil fertility over time when compared with traditional rotations, highlighted by increasing total C and N levels. This may be important in tropical regions, as tropical soils are sensitive to management because they lose carbon faster than their counterparts in temperate regions due to higher temperatures and rainfall [83] (Zomer et al. 2017). Besides improving the C and N status of soil under conservation practices, implementing doubled-up legume rotations can reduce pest and disease loads, increase beneficial insects, increase choices of plant based protein and improve overall resilience to climate change [84–86] (Rusch et al. 2013; Degani et al. 2019; Liu et al. 2020). This highlights the potential for CA principles and practices to contribute to climate adaptation in a region where temperatures are likely to increase. Higher yield and combined protein yield under CA compared to a CV system suggests that the negative impact of future climate change can be reduced by adopting the CA system over CV. Changes in phenology in future climate conditions create an opportunity to optimize management practices such as sowing date, row spacing and usage of cultivars with different maturities, which could further reduce the negative impact of temperature and enhance the benefits of CA [16, 87] (Naresh Kumar et al. 2016; Rodriguez and Roxburgh 2016).

### Potential implications for Mozambique

Recent studies have found, however, that especially for legumes such as pigeon pea protein content could be significantly reduced by elevated temperature and CO_2_ [99] thus signifying that nutritional quality could be severely impacted under future climate change scenarios. Other research at the global level, though focussed on wheat, has illustrated that though there may be benefits to wheat grain and protein yield under elevated CO_2_ scenarios this is largely offset by rising temperature and changes in rainfall [100]. This is particularly relevant for areas of low rainfall in SSA where nitrogen is a limiting factor. Adaptation options such as the introduction of genotypes that are adapted to warmer climates may improve both grain and protein yield, however, increasing grain yield does not necessarily improve protein concentration and thus concerted efforts need to be focused on this including for other crops [100]. Research relevant to Central and Northern Mozambique has shown that direct-seeded manual CA treatments performed better than conventional tillage, resulting in higher maize and legume yields based on direct yield comparisons. Moreover, improved drought tolerant maize varieties also outperformed the traditional control variety under different tillage treatments highlighting the benefits under a CA system [101]. Interestingly, alongside grain yield farmers’ also invariably preferred traits such as early maturing varieties and those that reduce pre and post-harvest losses and pest infestation [101]. Thus, inclusion of drought tolerant/early maturing varieties will further support sustaining yield and protein quality (particularly in nitrogen-limited systems). Research in Southern Africa, especially relating to sandy soils such as those in Northern Mozambique have highlighted that these are priority areas for CA systems and an integrated approach will be needed which includes soil carbon from multiple sources such as biomass from retention of crop residues, cover crops, trees, and the judicious use of mineral fertilisers thereby increasing the resilience of these systems and reducing soil fertility decline [102]. However, these inputs are often unavailable or unaffordable for many smallholder farmers, including those in parts of Northern Mozambique. 

Notwithstanding, constraints to usage (see [2] for an overview which is relevant to Mozambique), and the need to consider farmers’ priorities/local adaptation, crop diversification has been found to be low in some regions of Mozambique which can thereby increase the risk of yield losses due to adverse climatic conditions [88]. The potential delay in yield benefits under CA may be a deterrent for some farmers, however [89], Lalani et al., (2017) showed for the same district under study in Northern Mozambique that even among the poorest farmers (no external inputs used) using the same-crop mix; those practicing CA had a higher probability of breaking even and achieving a higher net return than farmers practicing conventional tillage, however, benefits in the short-term largely depend on crop-mix and opportunity cost of labour assumed. The projected rise in temperature under the various scenarios may also likely increase crop pest infestations in Mozambique [88] though less so with doubled-up rotations as noted [84] and results from Uganda suggest arguably appreciably lower under a CA system and when applied with push-pull technology [90] (Hailu et al., 2018). However, intercropping with three crops can increase pest infestation. To fully realise the benefits of CA, diversified intercropping systems, improved crop sequences (e.g. rotations), and suitable cultivars are therefore considered important for enhancing productivity, managing pests, and maintaining soil health. It has also been suggested that an increase in extreme weather events will also inevitably impact key local value chains/cause disruptions to food systems in Mozambique [91] (Macassa et al., 2021).

Oppewal and Da Cruz [92], has however, argued there is considerable scope to strengthen local value chains and increase domestic consumption of pigeonpea (majority exported in recent years and especially from Zambézia Province) in Mozambique, especially given the volatile export market. In neighbouring Malawi, for example, approximately half of the production is consumed locally, suggesting there could be scope for increasing domestic consumption in Mozambique and thereby help to improve food and nutrition security. In addition, there is potential of diversification towards other types of legumes, such as cowpea given local consumption is much stronger in Mozambique [92]. Charrua et al., 2021 [93] has also suggested that further incorporation of legumes into cropping systems within Mozabique will help to improve dietary diversity (especially if higher yielding) though a lack of research and investment severely impedes this. Moreover, better understanding of urban consumer preferences in Mozambique for cowpea/pigeonpea, for example, and improving marketing linkages (even with informal markets) will be beneficial for producers and consumers [93].^5^

Our results support those from neighbouring Malawi which have shown that growing a wide range of crops (e.g. maize intercropped/rotated with a legume) under a CA system will diversify cropping systems thereby increasing their resilience to climate change whilst contributing to improving food and nutrition security [94] (Muoni et al., 2024). Furthermore, the choice of system (CA as opposed to conventional) was found to be more important than varietal choice. It has also been argued, however, that shorter duration varieties are preferred in Mozambque as they are able to mature faster and that the usage of improved varieties (e.g. drought tolerant maize) is generally low [94] and that these in conjunction with CA would be particularly beneficial [88, 94, 95]. Nyagumbo et al. (2024) also showed in an on-farm trial assessing the performance of CA cropping systems in Sussundenga (Central Mozambique), that the largest yield gain relative to the ‘true farm practice’ was generated from CA cropping systems involving all three CA principles (i.e. CA maize-legume rotation with retention of crop reidues). Moreover, in total, 57% of the increase in maize yield was attributable to agronomic practices (e.g. timely planting) and 43% to the use of the three CA principles, though the comparison was compared to sole crop maize (as was [95]) and all treatments included the application of fertilisers/herbicides. As mentioned, this, however, further reinforces the relevance of basic agronomic practices as a stepping stone [16] and that future climate conditions may result in a change in phenology, requiring optimising management practices, which could further reduce the negative impact of an increase in temperature and more so under a CA system [87]. Most of these studies involving CA also invariably focus on two crops with the use of external inputs reinforcing the novelty of our approach and findings; exploration of multicrop interactions (i.e. at least three different crops)/locally specific practices (e.g. no external inputs etc.) and locally relevant crop mixes similar to those being practiced by farmers’ in the region [89].

### Limitations and future work

Crop choices and cultivation practices are also influenced by socio-economic and socio-cultural aspects. To gain a better understanding of these factors we agree with [13] Nhantumbo et al. (2017), who suggested that a whole- farm household analysis is required. One modelling approach could be the use of APSFarm, the whole farm/household configuration of APSIM (https://www.apsim.info/support/apsim-training-manuals/apsim-training-simlesa/apsfarm-simulations/) which is able to consider the labour and a number of plots of land/proportions devoted to each crop and can be modelled to see which proportions/mixes achieve a desired food security threshold. Although this study has attempted to simulate farmers practicing a full CA system i.e. more than two crops/diversity of crops, this does not take into the account the particular resource endowments of the farmer or multiple plots. Furthermore, whilst other research has modelled different CA practices (e.g. basins, dibble stick or animal ripper) in the region [13] (Nyagumbo et al. 2017), future research could simulate different CA practices taking a whole-farm modelling approach and one which also considers farmer typologies [13, 60] (e.g. Taore et al., 2017; Nhantumbo et al. 2017).

Anwar et al. [76] (2015) have also shown that higher future temperatures can contribute to reducing crop productivity primarily due to advanced crop phenology albeit in a different context and for different crops. This study was conducted with current cultivar traits and crop and soil management practices. In the future improved cultivars and crop management practices can enhance water retention and mitigate temperature stress, potentially altering the effect of CA and CV. It should also be noted that the model in this study was not extensively calibrated for extreme weather events such as drought and hot years which could be an area of future research.

We did not find measured data to obtain a better understanding of trade-off between grain yield and protein content. Because of this, we used a simplified approach to estimate the protein content provided in the study [47] conducted in and African context. Therefore, all the factors that affect grain yield will affect protein content, however, we note that the relationship between these two variables may change due to different G x E controls. Therefore, the interpretation of protein content in this study should be viewed in light of these limitations. The simulation setup in this study did not consider grain yield reduction that can occur by biotic stresses like weed infestations, insect pests and plant diseases as well as by pollutants like ozone or heavy metals if present in the study area.

## Conclusion

The results of this paper highlight the importance of rising temperature as temperature plays the most important role, responsible for almost 60% of the variance in the combined protein yield. We found that projected future trends illustrate that combined protein yields under both systems, Conservation Agriculture (CA) or conventional agriculture (CV), steadily decline under increasing temperatures; however, protein yield was always higher for CA relative to CV. The decline was more pronounced for cowpea and pigeonpea and less for maize. Protein yield was highest for cowpea and lowest for pigeonpea under both systems. Our results highlight the potential adaptive benefits of Conservation Agriculture for improving protein yield and overall productivity in maize-based cropping systems in sub-Saharan Africa (SSA) under changing climatic conditions.

### Policy and practice

Since 2017, Northern Mozambique (Cabo Delgado), among the poorest provinces in Mozambique, has been beset with armed conflict, which has affected more than 1.3 million people, further compounding vulnerabilities to climate shocks ([96] WFP, 2022; [97] UNDP, 2022). Building on farmers’ own adaptive strategies, Mozambique has prioritised farmer-led, co-developed Conservation Agriculture. This approach is supported through complementary initiatives that provide access to inclusive finance, secure land tenure, and farmer-centred extension, including climate services, with particular attention to women, youth, and internally displaced people in conflict-affected areas ([88, 96, 97]; CIAT; World Bank 2017). Equally, there is a focus on strengthening local food systems and increasing value-added chains nationally [96]. There thus exists considerable scope for ‘underutilised indigenous and traditional crops’ to contribute towards the development of low-input systems [98] **(**Mabhaudhi et al., 2020). In tandem, strengthening local seed systems, input/output markets for neglected and underutilised crops will likely benefit local food systems (e.g. focus on domestic markets/consumption of cowpea and pigeonpea) and thereby improve food and nutrition security [98].

## Supplementary Information

Supplementary Material 1.

Supplementary Material 2.

## Data Availability

All data generated or analyzed during this study are included in this article. All data will be available on request to corresponding author.

## Footnotes

1 ‘Underutilised indigenous and traditional’ crops often referred to as those that may have less developed value chains/untapped potential. This may vary based on geographic/socio-ecoomic settings. See Mabhaudhi et al. (2019) for a comprehensive overview.

2 It should be noted that we modelled maize, cowpea, and pigeonpea in various intercropping systems, with all crops grown on the same plot of land during the same season. Specifically, two systems were modelled: one with maize and cowpea, and another with maize, cowpea, and pigeonpea.

3 To calculate protein yield the nutrient composition (g/100 g) was multiplied by the yield (e.g. 21.7/100 multiplied by yield = amount of protein for that specific yield). We calculated protein yield for the various crops per year/system individually and then combined these i.e. ‘combined protein yield’ which is based on the specific crop mix so if it is maize, cowpea, pigeonpea (this would be protein yield of each of the crops in year x summed together and so on).

4 The socio-economic pathways are in direct alignment with our scenarios T0-T4. i.e. T0 = SSP1-1.9 (i.e. optimistic scenario where CO_2_, emissions are cut to net-zero by around 2050 with warming hitting 1.5 °C but then dipping back down and stabilizing around 1.4 °C by the end of the century). T1 = SSP1-2.6 the next-best scenario, global CO_2_ emissions are cut severely, but not as fast, reaching net-zero after 2050, temperatures stabilize around 1.8 C higher by the end of the century. T2 = SSP2-4.5 a “middle of the road” scenario. CO2 emissions hover around current levels before starting to fall mid-century, but do not reach net-zero by 2100. In this scenario, temperatures rise 2.7 °C by the end of the century. T3=, SSP3-7.0: emissions and temperatures rise steadily and CO2 emissions roughly double from current levels by 2100. By the end of the century, average temperatures have risen by 3.6 °C. T4 = SSP5-8.5: This is a future to avoid at all costs. Current CO2 emissions levels roughly double by 2050. By 2100, the average global temperature is a scorching 4.4 °C higher.

5 Cooking classes in Zambia with smallholder farmers have been initiated to make pigeonpea more appealing and reduce dependence on exports to India. See URL: https://cgspace.cgiar.org/server/api/core/bitstreams/79d57921-a644-4f24-b495-80e5f834d22c/content.
