# Supplementary material for: Simulations using APSIM suggest that Conservation Agriculture sustains protein yield under changing climate dynamics in Northern Mozambique
Source: BMC Plant Biol. 2025 Nov 13;25:1556. doi: 10.1186/s12870-025-07418-5 (PMC12613769; doi:10.1186/s12870-025-07418-5)
Supplement: Supplementary file 1 — Supplementary Material 1. [file 12870_2025_7418_MOESM1_ESM.docx]

**Supplementary Figure 1 A**

Inputs/Environment

Specific Leaf Nitrogen

Leaf initiation rate

Leaf appearance rate

Root elong rate

RUE

Parameters

Maturity

Flowering

Leaf Number

T Leaf Number

Floral initiation

Phenology

Root depth

Water stress

Leaf area

Senesced leaf area

Leaf area

Expansion

Fraction of radiation interception

CHO partitioning

T supply

TE_actual

T demand

RUE_actual

Resource capture

Grain number

Biomass around anthesis

CGR_water

CGR_rad

CGR_actual

Biomass

Grain N

CHO translocation

Biomass during grain filling

Grain set

Grain size

Grain yield

Grain fill

Yield

**Supplementary Figure 1 B**

Soil N

N fertilizers

Grain number

CHO partitioning

Environment

N demand per grain

Critical Specific leaf Nitrogen

Root elong rate

Parameters

Max N uptake rate

Minimum Stem N %

Senesced leaf area

Leaf area

Stem mass

Expansion

N Supply

Leaf N demand

N uptake actual

Stem n demand

Resource_capture

Specific leaf Nitrogen

Stem N%

N Biomass

Grain N demand

Grain set

N translocation

Grain fill

Yield

Grain N
